# The existence and importance of patients’ mental images of their head and neck cancer: A qualitative study

**DOI:** 10.1371/journal.pone.0209215

**Published:** 2018-12-31

**Authors:** Heidi Lang, Emma F. France, Brian Williams, Gerry Humphris, Mary Wells

**Affiliations:** 1 School of Nursing and Midwifery, University of Dundee, Dundee, United Kingdom; 2 Nursing, Midwifery and Allied Health Professions Research Unit, Faculty of Health Sciences and Sport, University of Stirling, Stirling, United Kingdom; 3 Social Dimensions of Health Institute, University of Dundee, Dundee, United Kingdom; 4 School of Medicine, University of St Andrews, St Andrews, United Kingdom; Iranian Institute for Health Sciences Research, ISLAMIC REPUBLIC OF IRAN

## Abstract

**Objectives:**

To explore the existence and importance of mental images of cancer among people with head and neck cancers with a focus on the perceived origins and meaning of mental images, their development over time, and their relationship to illness beliefs.

**Methods:**

A longitudinal qualitative study consisting of 44 in-depth semi-structured interviews with 25 consecutive, newly-diagnosed head and neck cancer patients. Participants were invited to draw their images during the interviews. Follow-up interviews occurred after treatment completion. Analysis drew upon the principles of Interpretative Phenomenological Analysis (IPA).

**Results:**

Many participants had mental images of their cancer which appeared to both embody and influence their beliefs about their illness, and affect their emotional response. For those who held them, mental images appeared to constitute an important part of their cognitive representation (understanding) of their illness. For some, their images also had a powerful emotional impact, being either reassuring or frightening. Images often appeared to originate from early clinical encounters, and remained fairly stable throughout treatment. Images could be conceptualised as ‘concrete’ (the perceived reality) and/or ‘similic’ (figurative). Patients’ images reflected the perceived meaning, properties or ‘intent’ of the cancer–that is beliefs concerning the disease’s identity, consequences and prognosis (likelihood of cure or control).

**Conclusions:**

People with head and neck cancer may develop a mental image of their disease, often generated early within clinical encounters, which can both reflect and influence their understanding of the cancer. Such images tend to be stable over time. We theorise that careful use of images in early consultations could avoid or minimise some distress, including fears of outcome or recurrence. Concrete or similic images and language could be employed later to change perceptions and reduce distress.

## Introduction

Head and neck cancers (HNC) affect the most visible parts of the body and have a considerable physical and psychosocial impact [1]. Levels of anxiety, depression, and distress in this population are significant [2, 3]. Continued impairments to quality of life [4] and fears of recurrence often persist beyond treatment [5, 6]. Recent work suggests people’s understandings of their HNC and treatments are an important target for interventions to improve psychological outcomes [7]. There is increasing evidence that illness beliefs in people with cancer explain significant variance in many outcomes including psychological adjustment [8], symptom experience [9], distress post-treatment [10], fear of recurrence [11] and quality of life [12] and that these beliefs may be modifiable [13].

Illness beliefs may be embodied in visual form in people’s internal, mental images of their disease [14]. Such images can powerfully affect the psychological experience and impact of illness [15], self-care behaviours and disease outcomes,[14, 16] and predict the preventative behavioural intentions of healthy people [17]. For example, in Broadbent *et al*.’s research [14], people’s drawings of the damage to their heart following a myocardial infarction, and their associated illness beliefs, predicted recovery better than clinical indicators of actual damage. Eliciting people’s illness beliefs and elucidating the role of mental images in these constitutes an important first step in developing interventions aimed at modifying illness beliefs and associated mental images in order to enhance patient outcomes.

No research that we know of has explored people’s internal mental images of their HNC. Limited existing work exploring people’s images of their breast [18] or other types of cancer (predominantly osteosarcoma, leukaemia and lymphoma) [19] has indicated the potency of those images in terms of emotional distress [18, 19]. Using qualitative interviews alongside drawings, Harrow *et al*. [18] found women’s mental images of their breast cancer embodied positive and negative beliefs, reflecting their perceptions of cancer’s physical identity, consequences in terms of behaviour within the body, and likelihood of spread or recurrence; these perceived properties of their cancer were linked to prognostic anxiety and fear of recurrence. An educational intervention trial [19] which involved showing adolescents samples of their cancer tissue found dramatic changes in their mental images of, and attitudes towards, their cancer. After the intervention, adolescents’ original mental images appeared to be replaced with the comparatively less threatening images of the cells they had seen via a microscope, which positively affected their emotional response to the disease [19].

Previous research examining illness beliefs in people with HNC [12, 20] has used quantitative measures only, however, qualitative study designs are optimally suited to exploring in-depth the meanings and significance of patients’ experiences and perceptions. We conducted a longitudinal qualitative study exploring the existence and importance of people’s mental images of their HNC. The study design was informed by a qualitative phenomenological perspective [21] and underpinned by the theoretical framework of the self-regulation model [22, 23]. The model proposes that in the context of illness three interactive psychological processes are consciously engaged: 1) interpretation and the generation of an ‘illness representation’, 2) the implementation of coping strategies, and 3) appraisal of their efficacy. The concept of an ‘illness representation’ comprises a cognitive representation consisting of illness beliefs along five dimensions–identity, cause, consequences, timeline, and cure/control—and an emotional response (emotional representation). These five dimensions have been extensively investigated empirically, and in this study were used to explore and classify participants’ illness beliefs. The aim of our study was to explore in detail the existence and importance of mental images of cancer among people with HNC in terms of the perceived origins and meaning of mental images, their development over time, and the relationship of their mental images to illness beliefs.

## Methods

### Study design

A longitudinal qualitative design was adopted to enable an in-depth exploration of participants’ mental images of cancer, illness beliefs and experiences and how images and beliefs developed over time. Participants were invited to take part in two semi-structured interviews, the first prior to or during early treatment, the second at least one month after treatment completion.

### Sampling and recruitment

Patients were recruited through the oncology departments of two Scottish hospitals. Consecutive sampling was employed (purposive sampling was not feasible due to the limited population available). A target sample size of twenty-five was set based on the projected number of new patients within the study timeframe and an estimate that half of eligible patients would consent to take part. All newly-diagnosed adult HNC patients were approached providing they were English-speaking, and able to give consent. Patients undergoing palliative treatment were not excluded. Clinic staff approached eligible patients after their attendance at a scheduled appointment. An information sheet and invitation letter was given to prospective participants, and clinic staff obtained consent for the researcher to telephone them to discuss the study further, and arrange interviews. Clinicians approached 36 patients, 25 of whom agreed to participate. All participants signed a consent form on the day of interview. NHS Fife and Forth Valley Research Ethics Committee approved the study (approval number 08/S0501/5).

The sample comprised 17 men and 8 women aged 31–79 years old (mean age 57). Laryngeal cancer was the most common type, and most participants had undergone multimodal treatment. Participants’ demographic and treatment details are shown in Table 1.

**Table 1:** Sample characteristics.

		Number
**Age**	30–39	2
	40–49	5
	50–59	7
	60–69	5
	70–79	6
**Gender**	Male	17
	Female	8
**SIMD ranking** (quintiles) **(**The Scottish Index of Multiple Deprivation (SIMD) combines data on seven domains including income, education, employment and crime to produce a relative ranking of all Scottish postcode areas from most to least deprived.[24])	1: most deprived	6
	2	3
	3	4
	4	7
	5: least deprived	5
**Participation Level**	Two interviews	19
	One interview	6
**Primary disease site** (self-reported)	Larynx	8
	Tongue	3
	Tonsil	3
	Oral cavity	3
	Neck	4
	Nasopharynx	1
	Throat	3
**Treatment regimen**	Radiotherapy only	6
	Radiotherapy and surgery	5
	Radiotherapy, chemotherapy and surgery	9
	Radiotherapy and chemotherapy	5

### Data collection

Interviews were guided by a semi-structured topic guide to ensure topics relevant to the research questions were covered while also enabling participants to raise unanticipated issues. A copy of the ‘Time 1’ (T1) and ‘Time 2’ (T2) topic guides are included in S1 File. This study was phenomenological in approach—the focus of the interviews was on the subjective experiences and meanings of mental images amongst HNC patients. The main topics addressed were: participants’ illness beliefs in terms of Leventhal’s five dimensions—identity, cause, consequences, timeline cure/control; their understanding of planned treatments; and the existence, meaning and perceived origins of any mental images of the cancer. Second interviews were informed by a summary of the first interview, alongside the T2 topic guide, to enable exploration of the stability/instability of beliefs and images and issues raised at the first interview. Rapport was built with participants through informal conversation prior to commencing the interview. During the interview participants were invited to sketch any mental images of the cancer using white paper and coloured pens and pencils, while verbalising the meaning of their images. Field notes were compiled after the interview.

Interviews were carried out by HL, a female postgraduate student from a social sciences background (psychology/sociology), as part of her doctoral thesis. The interviewer was not a clinician and had no involvement in patient care. Her first contact with participants was via an introductory telephone call during which the purpose and requirements of the study were discussed. Prior to the interviews she underwent training in qualitative interviewing on sensitive subjects, consulted with clinicians experienced in the HNC field, and conducted mock interviews.

In total 44 audio-recorded interviews were conducted lasting between thirty minutes to three-and-a-half hours, from March 2008 to June 2009. The location of the interview was the participant’s choice, usually taking place in their own home, but on 10 occasions in a private room or café within the recruiting hospitals. The participant’s spouse was present at four interviews with three participants. Nineteen participants completed both interviews. Three died during the treatment period, and three withdrew prior to the second interview (one due to the recurrence of his cancer, one without giving a reason, and the third due to scheduling difficulties). The time between first and second interviews ranged from three to nine-and-a-half months (mode: 5 months).

### Data analysis

Interviews were transcribed verbatim. All authors were involved in analysis and interpretation: HL took primary responsibility, MW and BW coded a sub-sample of transcripts and were involved in discussing and analysing all interviews, and EF read all transcripts and conducted further analysis. GH read transcripts and edited the manuscript. Each transcript was analysed by at least two authors working independently. A subset of transcripts was scrutinised by a further two authors to establish the validity of the interpretation, and themes were developed and refined collaboratively. Four of the five authors (BW, MW, EF, GH) are experienced qualitative researchers.

Thematic analysis was guided by the principles and procedures of Interpretative Phenomenological Analysis (IPA) [21] which involves six stages: (1) initial coding of the first transcript, (2) making connections between preliminary codes/themes, (3) constructing a master list of themes, (4) analysing further transcripts by repeating stages 1 and 2, (5) intensive analysis of individual themes, and (6) investigating patterns, connections and tensions amongst the body of themes. Analysis was longitudinal and cross-sectional, informed by Leventhal’s [22, 23] concept of illness representations and the dimensions of identity, cause, consequences, timeline, and cure/control. However, codes and themes were mostly derived inductively from the data. Analysis of participants’ images focused on exploring the subjective meaning of the images as described by the individual participants. NVivo 8.0 computer software was used to organise and manage the data. Participants did not provide feedback on the findings; however the interpretation of the T1 interview data was verified with participants during T2 interviews to ensure key aspects had been correctly understood.

## Findings

Many participants generated mental images of their cancer. Such images appeared to reflect and influence participants’ perceptions of their illness, and thereby impact their illness experience and, for some, their emotional response. A number of factors seemed to influence the development of particular mental images, including visual and verbal images provided by clinicians. Interview excerpts are labelled with the participant’s pseudonym, age group, cancer type and if it was a first (T1) or second interview (T2).

### Existence and nature of mental images of HNC

**Fig 1:**
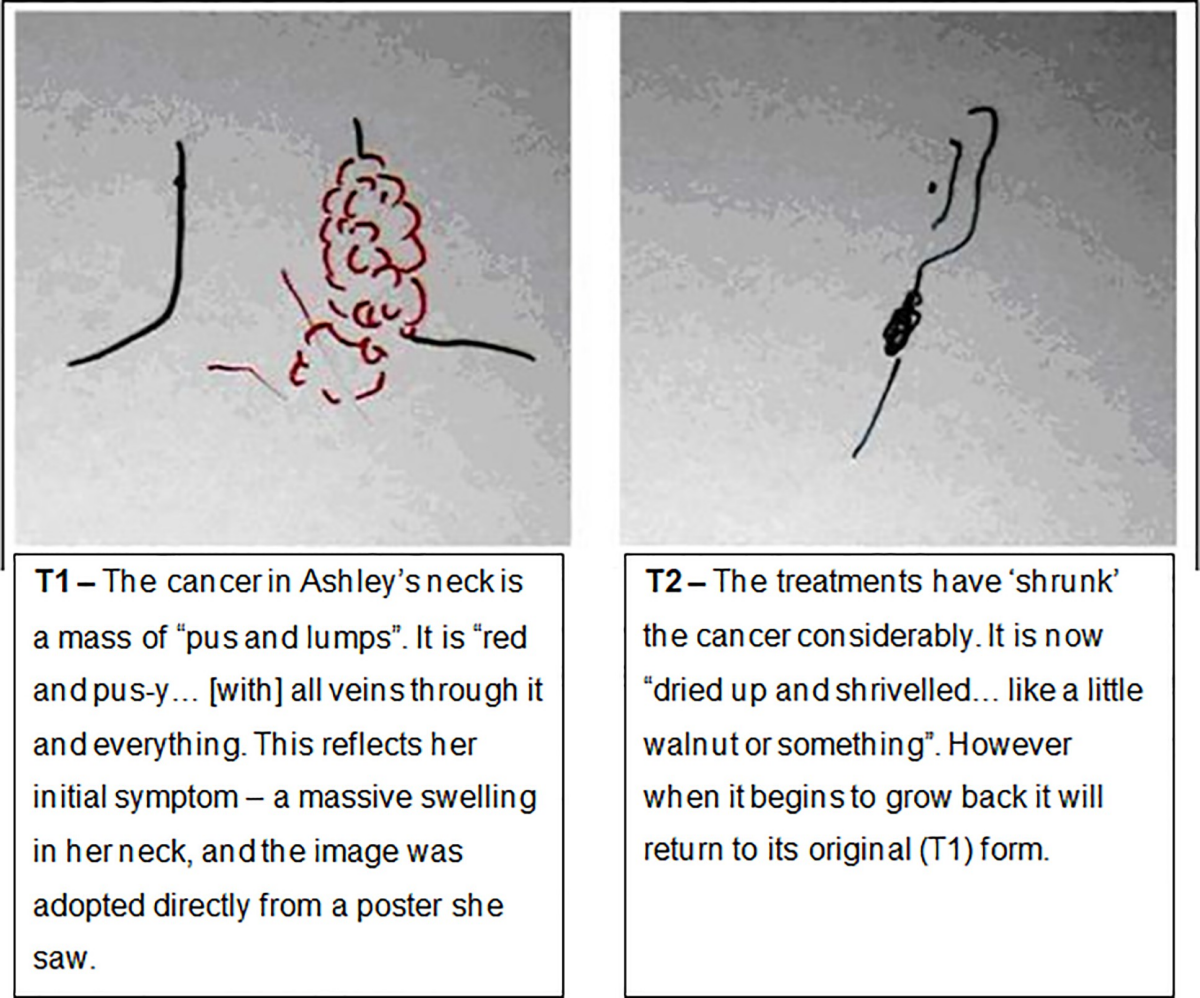
Ashley, T1 and T2, Cancer in the neck. The T1 drawing shows how Ashley perceived her cancer at diagnosis. The T2 drawing represents the cancer post-treatment.

**Fig 2:**
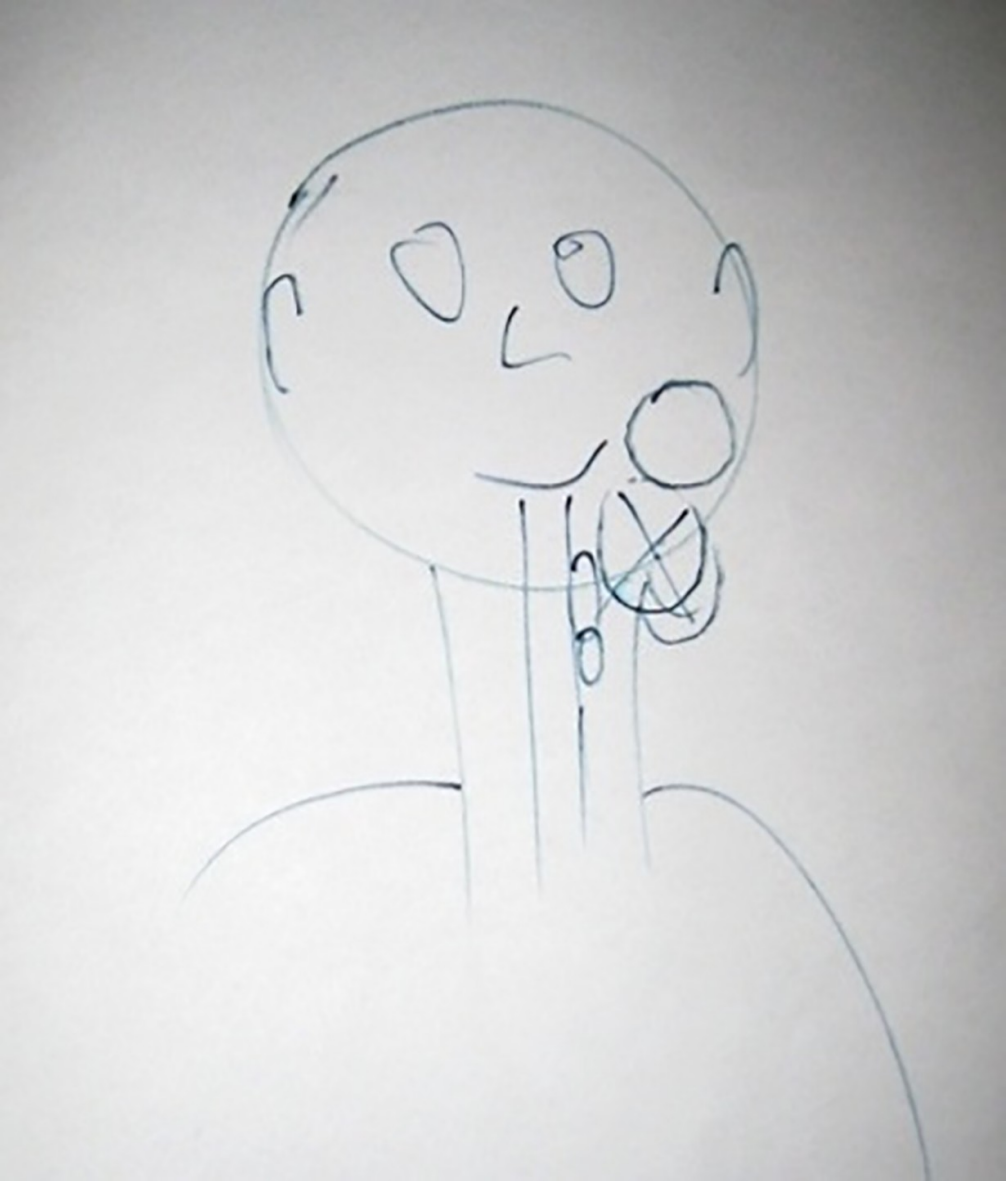
Alan, T1, Nasopharyngeal cancer. Drawing shows palpable lump in neck.

**Fig 3:**
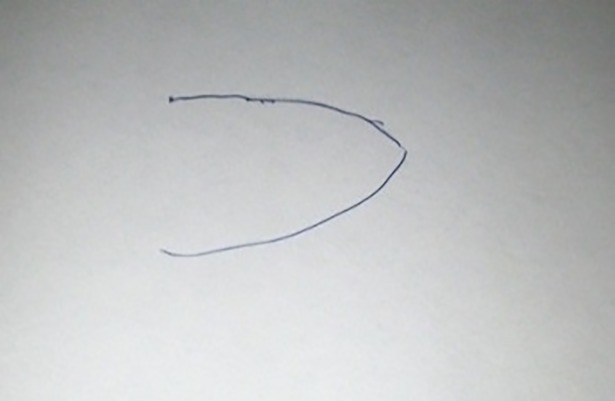
Brian, T2, Tongue cancer. Size and shape depicted.

**Fig 4:**
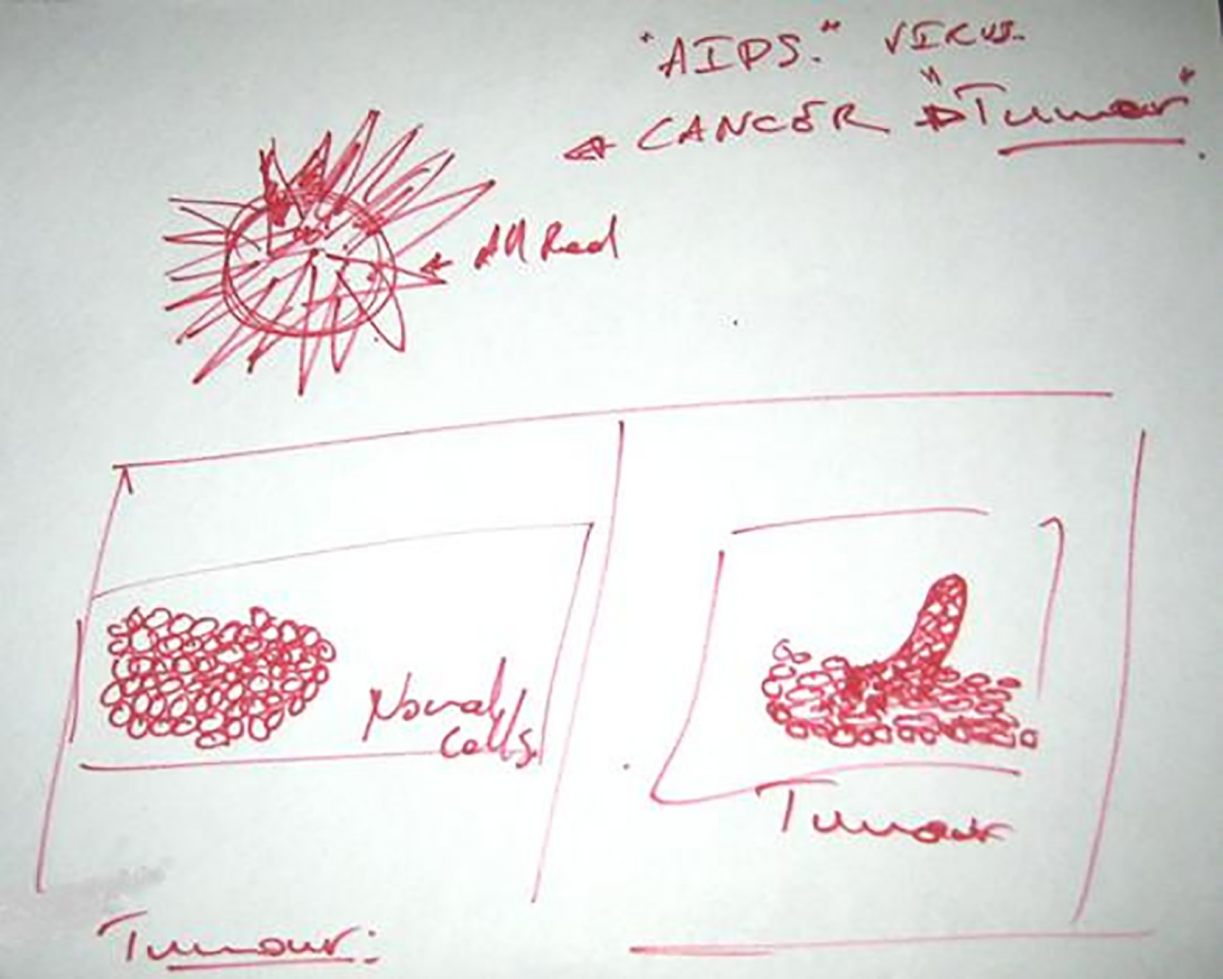
Jean-Claude, T1, Tumour in the oral cavity.

**Fig 5:**
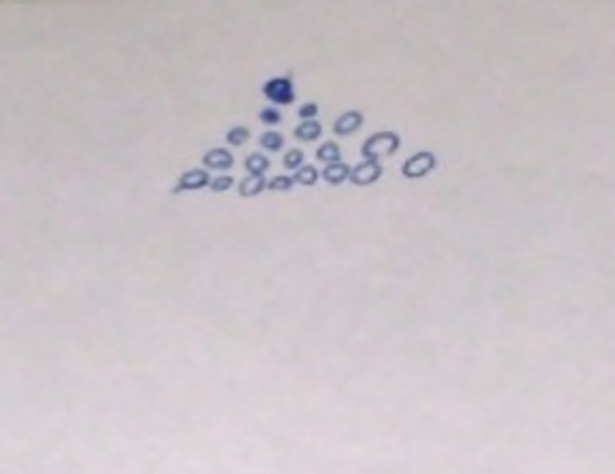
Jean-Claude, T2, Cancer cells multiplying.

**Fig 6:**
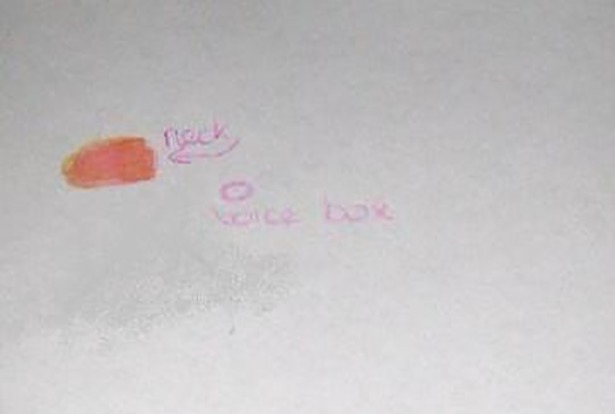
Katrina, T1, Lump in her neck.

**Fig 7:**
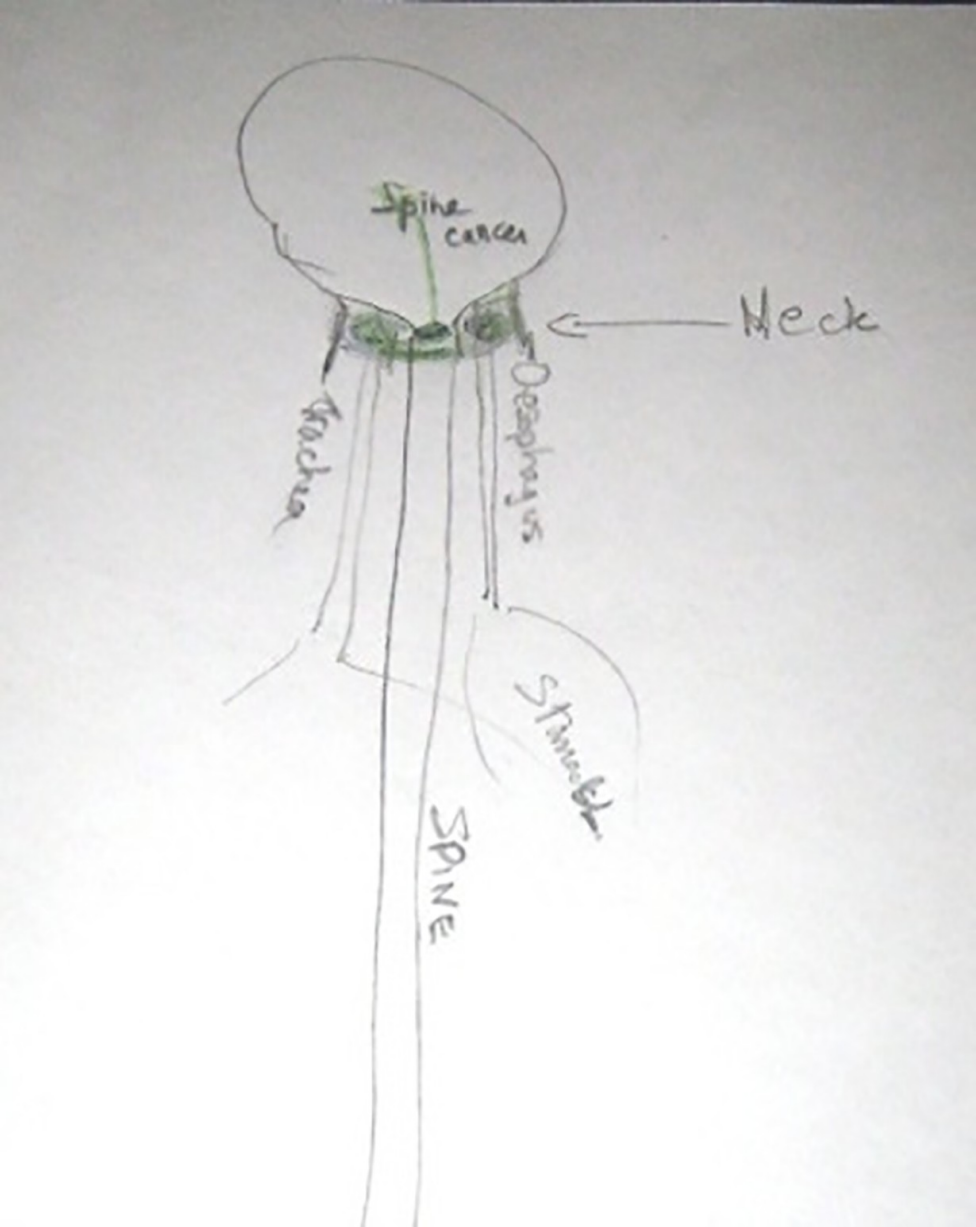
Nell, T1, Neck cancer.

**Fig 8:**
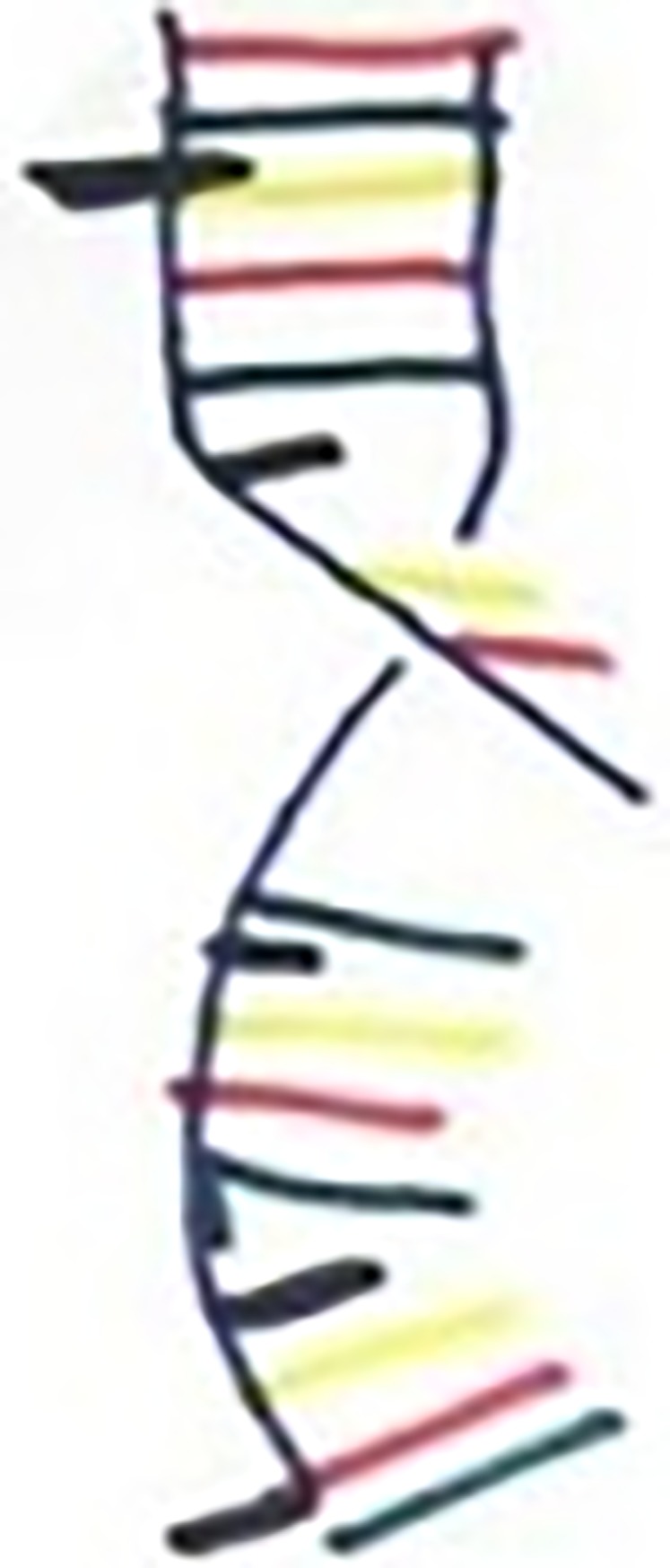
Norman, T1, Cancer as corrupt DNA.

**Fig 9:**
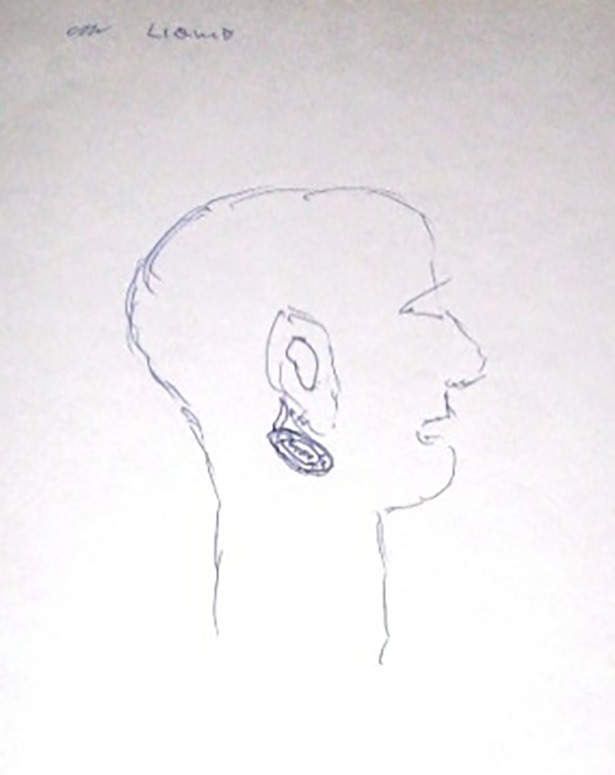
Scott, T1, Cancer in neck cyst.

Most participants (17/25) claimed to have, and could express verbally, a mental image of their cancer. Table 2 details the range of images described verbally by participants. Images varied in their complexity and level of abstractness. Only eight participants agreed to draw their image, these drawings are presented in Figs 1–9. The majority of participants who declined to draw their image cited a perceived inability to re-create the image accurately, but a few gave no reason. Younger participants were more likely to agree to draw an image–half (7/14) of those aged 30 to 59 did so, compared to only one of the eleven participants aged 60 to 79. Most participants’ images of the cancer were stable between their first and second interviews. However, some of the images evolved reflecting the perceived progress of the disease, impact of treatments, or incorporation of new information. Fig 1, below, demonstrates the evolution of one participant’s mental image of their cancer.

**Table 2:** Participants' verbally conveyed mental images.

Participant (age)	Example verbal images	Drawing produced?
Alan (30s)	‘I just see mine as a rough ball, a sort of ball-shaped rough skin.’ (T2) ‘It’s all blood, any tissue you can say everything’s red, it’s the same. To me it would just be a continuation of the tissue that’s already there. You see a bit of surgery, you see someone cut open, they’re red, be it within the cheek, within the leg, within the stomach. It’s all red.’ (T2)	Yes–Fig 2.
Ashley (40s)	‘It was just red and pus-y […] It was just like, all veins through it and everything. [..] I can only visualise it as I saw it there [on the poster], and that’s because you’d imagine anything like that to be red, wouldn’t you? Because it’s to do with your veins and bloods. [..] like an open wound, you’d expect it to be red’ (T1) ‘Pulsing in the corner [. . .] malevolently’ (T2) ‘You just imagine it as dried up and shrivelled at the moment. [..] Like a little walnut or something’ (T2)	Yes–Fig 1.
Brian (50s)	‘His [the consultant’s] drawing was just a sort of 5cm by about 3cm growth and that’s just really how I looked at it’ (T1) ‘He [the consultant] showed me dimensions of it and I was able to contain it in that bit. […] It was much better, much easier to focus on something like that than on something that was more invasive. Right, it [the cancer] is in that little box. I can deal with that little box, it is so much easier to deal with’ (T2)	Yes–Fig 3.
Jean-Claude (30s)	Quotes cited in preceding text.	Yes–Figs 4 and 5.
Katrina (40s)	‘It would be like a blob, it would be a skin colour. […] Maybe nearly as big as a grape on the voice box and the tongue. [..] Like a squashy orange.[..] And if you touched it, it would have moved about but not all over just in the area […] Like skin that shouldn’t have been there, that was maybe, err how can I explain? Marshmallow’ (T1)	Yes–Fig 6.
Nell (70s)	‘It’s basically just a mass, plus this one bit at the top of the bones’ (T1) ‘A reddish mass’ (T1) ‘I still visualise it as almost a heaving mass. Because it’s growing fast, […] I see it as this thing forever on the move. […] Pulsing maybe’ (T1) ‘They [the cancer cells] should be black and evil but I don’t think they are. […] Maybe it’s not that colour, maybe it’s white, or sort of rather colourless’ (T1)	Yes–Fig 7.
Norman (50s)	‘It shows that light has been absorbed and not reflected, you know, like a dark star. Don’t go near it or else you’ll never get out, you know, like a black hole. It doesn’t radiate anything. It just absorbs, it takes in and devours and that’s why it would be black.’ (T1) ‘It’s the body turning against me. The DNA’s been corrupted and it’s slowly poisoning me and creating tumours and eventually well, it’ll do its job and that’ll be it.’ (T1)	Yes–Fig 8.
Scott (40s)	‘My cyst would just be two circles; one inside the other with a tail. […], well the branch where it was the branchial. […] and it’s sort of double walled and it has a sort of tail attached there where your branchials had been there, sort of down there. And that’s liquid and all your gore. And the cancer cells were found in here.’ (T1)	Yes–Fig 9.
Steve (50s)	‘The bad cells are growing and taking over an area, destroying whatever it’s affecting. I’m assuming it’s attached to fleshy bits, and whether it’s eating away […] spreading, killing off good cells” (T1)	No
Alasdair (50s)	‘I have this kind of image of a chain-reaction of cells that are dividing and basically like weeds developing in a vegetable patch—they’re spreading quite quickly. But then in, in order to eradicate these weeds, it’s also in some cases necessary to eradicate the vegetables too’ (T1) ‘Little devil’ (T2)	No
Eric (70s)	Quote in subsequent text.	No
William (60s)	‘It must be like black ink spreading, you know. That sort of thing, some sort of thing spreading […] I think it may be some sort of fluid or something that’s spreading.’ (T1) “I think cancer is a sort of wastage [..] maybe there’s a weak part of the body and somehow or other it gets attacked by this cancer.” (T1)	No
Cathy (40s)	‘Kind of evil creature’ (T1) ‘a big, deep, dark gorge,’ ‘wee germ bug things,’ ‘ferociously […] eating away my tongue’ (T2) ‘I could actually see it. […] You could just see this kind of black. The longer it was left, you could see it getting deeper and deeper, almost like eating the tongue away almost’ (T2) ‘The wee bug things represented to me the bad cells, the cancer cells, and it was almost like they were just, “ha, ha, ha.” The black gang had kind of jumped onto the good cells or the good guys and bashed them a bit and that’s what they’re kind of laughing at’ (T2)	No
Lewis (70s)	‘A wee bit sticking out [..] like a piece of gristle or something’ (T1) ‘I just assumed it was just like a vegetable growing. Once it starts, it expands’ (T2)	No
Christopher (50s)	‘Like a sausage without a skin with lots of blood vessels’ (T1) ‘Making base camp and setting out their nasty stall and they end up taking over’ (T1) ‘Highjack[ing] your body’ (T2) ‘Like a dandelion root will grow in between paving stones [..] if it meets something stronger it will move round it, maybe that’s why it’s so deadly because they grow around something rather than try and fight it, it will just try and find another way round it’ (T2)	No
Albert (70s)	‘The roots would get longer, bigger […]. You can see a tree or a flower with a little root, growing bigger and bigger and bigger. Take a hold […]. The bigger it got the harder it is to get it removed’ (T1)	No
Jill (60s)	‘Rotten flesh I suppose. Something going off. […] A piece of rotten meat [..] Something you’d throw in the bin’ (T1)	No

### Concrete versus similic images

Participants’ mental images could be conceptualised as ‘concrete’, representing the perceived reality of the cancer, or ‘similic’ i.e. figurative in nature. Some participants held both concrete and similic images. Concrete images represented beliefs regarding the identity of the cancer in physical terms, and were therefore significant in a person’s understanding (cognitive representation) of their disease. The colour, size, location, and shape of the cancer were the most frequently reported attributes in these images.

Similic images often reflected participants’ broader understandings of the physiological processes involved in the cancer and treatments. An underlying concrete image could also involve similic elements, for instance, Alasdair likened the fast-growing cancer cells to weeds:

Cancerous cells are those which divide faster than other normal cells. So I have this kind of image of a chain-reaction of cells that are dividing like weeds developing in a vegetable patch, they’re spreading quite quickly. In order to eradicate these weeds, it’s also in some cases necessary to eradicate the vegetables. (Alasdair, 50s, throat cancer, T1)

A few participants, such as Eric, conveyed images that were solely similic. Eric’s description indicated that he did not perceive of his cancer as a discrete entity (like a tumour):

It strikes me as just a lot of dust flying around in the air and it’s all nasty. It’s like the weed-killer out there that I use in the work. (Eric, 70s, tonsils, T1)

Similic images conveyed in figurative language—often but not solely based on analogies to plants, vegetables or meat (examples in Table 2)—expressed perceptions and emotions about the properties of cancer, such as size, potency, behaviour (beliefs regarding disease consequences), and hence its responsiveness to treatment (beliefs regarding cure/control).

### Mental images of the appearance and characteristics of cancer

Eleven participants described the cancer’s colour; they described it as like other body tissues or blood, the colour black, or for one participant, as white on the surface and blood-coloured underneath. Perceptions of colour could be literal or figurative: a few people had actually seen their cancer or overheard health professionals or relatives describing its actual colour, others only imagined the colour. Those who spoke figuratively, and described the cancer as the colour of other body tissues, tended to see the cancer as part of their own body as opposed to an invasive foreign entity, for instance:

Any tissue you can see—everything’s red, it’s the same. To me it would just be a continuation of the tissue that’s already there. (Alan, 30s, nasopharynx, T2)

Two participants spoke figuratively about their cancer as black, this seemed to express their negative feelings about cancer as a disease, and perceptions of its virulence (i.e. their cure/control beliefs). For example, here Norman conveys his feelings about his incurable diagnosis:

It shows that light has been absorbed and not reflected, you know, like a dark star. “Don’t go near it or else you’ll never get out,” you know, like a black hole… It doesn’t radiate anything. It just absorbs, it takes in and devours and that’s why it would be black. (Norman, 50s, throat cancer, T1)

Participants’ descriptions of their cancer’s size and location tended to be concrete and were linked to beliefs about the disease’s consequences and the potential to cure/control it. Larger cancers were perceived as more established/advanced, therefore more severe and difficult to treat. A small-sized tumour tended to be perceived as having a better prognosis:

But it must be quite small because they can’t locate it with the biopsy, and with the camera and so on. So that again is relatively hopeful. (Alasdair, 50s, throat cancer, T1).

Participants’ understanding of the anatomical location of the cancer also affected their perceptions of the consequences in terms of the likelihood of the cancer spreading, and therefore also influenced their prognostic beliefs (cure/control). The belief that HNCs were less serious than cancers of specific organs was reasonably common among participants:

That’s why I’m not really down about it. If it was in my liver or kidneys or anywhere you know, bowel or anything like that, to me that’s serious cancer. (Andrew, 40s, tonsils, T1)

There were exceptions: two participants feared the cancer would spread to their brain.

Many participants described the cancer and its form or shape simply and literally as a ‘lump’, ‘bump’, ‘mass’, ‘spot’ or ‘round’. A few participants used more detailed visual language:

Like a dandelion root will grow in between paving stones […] so I can imagine that’s something similar. If it meets something stronger it will move round it, maybe that’s why it’s so deadly because they grow around something rather than try and fight it. (Christopher 50s, oral cavity, T2)

For some participants, the shape was related to the cancer’s perceived properties: in Christopher’s case, cancer’s perceived ability to circumvent obstacles made it deadly. Beliefs concerning shape were often related to the perceived ease or difficulty of treating it, e.g. roots were seen as more invasive and difficult to excise. Those who saw the cancer as having a discrete shape or as being ‘contained’ tended to perceive this as easier to treat, and so less dangerous. For instance, Scott felt reassured that his cancer would not spread elsewhere in his body because it was enclosed within a cyst in his neck:

In the end it doesn’t matter if it is the size of a pea or the size of a football, if it’s encapsulated within the cyst and it’s not migrated from the area it doesn’t make any difference. (Scott, 40s, neck, T1)

### Mental images of how cancer behaves

Participants’ mental images tended to represent cancer as either destroying or competing with the body and its structures for space (one alternative perception was expressed by Norman who saw cancer as his own body’s DNA attacking itself rather than cancer as a third party attacking his body). These contrasting perceptions appeared to be related to the participant’s perceived prognosis (beliefs regarding consequences) and likelihood of recovery (cure/control beliefs). The destruction of body tissues by the cancer was presumed to be irreversible, whereas participants with ‘competing’ images perceived that obstructed organs or structures would return to normal after treatment, for example:

The fact that’s [the tumour’s] now shrinking a little, it’s allowing me to breathe. That would suggest it’s retreating back to its space from whence it came allowing what should be there to expand back into its normal shape again. (Alan, 30s, nasopharynx, T1)

This suggests that the destroyer image was more frightening than the competitor image for many participants.

### Origins and impact of visual communications

Numerous external sources were identified as influencing participants’ mental images, and thus how they felt about their cancer and prognosis. Sources included discussions with clinicians and other health communications, e.g. posters, patient leaflets, and books. Images arising from clinical encounters included descriptive visual language used by clinicians; clinical images such as magnetic resonance images, computerised tomography scans, X-rays, and internal bodily images seen or described during naso-endoscopy examinations; and clinicians’ diagrams and drawings of the cancer. Information derived from clinicians and other medical sources was most trusted by participants.

For participants who did not have a pre-existing image of their cancer, external image sources appeared to influence the generation of a mental image, for instance: ‘I was at the doctor’s surgery and the poster [of a tumour] was on the wall. I saw that and thought, “Oh, that’s what it is!”‘ (Ashley, 40s, neck, T1). For participants who already had a mental image of their cancer, this could be reinforced by exposure to congruent external imagery, or result in modification or replacement of the existing image. For instance, Jean-Claude stated that prior to the first interview his mental image had been a ‘red ball with loads of spikes coming out’, (shown at top of his drawing in Fig 4), an image similar to the HIV virus in a 1980s health campaign. He associated this image with dying:

There would be this picture of the cell, the big red cell with spikes and you think “Oh my God, this is it, we’re all going to die!” (Jean-Claude, 30s, oral cavity, T1)

His newer, more literal image of ‘abnormal cells of tumour,’ (shown at bottom of his drawing in Fig 4) came from a patient information leaflet which challenged how he had visualised cancer: ‘I realised maybe I wasn’t thinking about it the right way.’

For the majority of participants, visual or verbal images of the cancer from external sources were highly influential on their mental image and overall cognitive representation of their cancer, but did not necessarily elicit a strong emotional response. However, for a few participants such images had a powerful emotional impact, either reassuring or frightening them, by communicating positive or negative messages about their cancer’s treatability (i.e. the images affected their cure/control beliefs). For example, a consultant’s sketch of his tumour provided Brian with a less threatening representation of it and so reduced his fear of the cancer spreading:

He showed me dimensions of it […] And it was much better, much easier to focus on something like that than on something that was more invasive. “Right, it is in that little box, I can deal with that little box.” It is so much easier to deal with. (Brian, 50s, tongue, T2)

In contrast, a clinician saying radiotherapy would be necessary ‘to get the roots’ of his cancer frightened Albert, who took the figurative language ‘roots’ literally. Until then he had been relatively confident of a good prognosis post-surgery.

Another important source of cancer imagery, not originating from clinical communication, was the ability to directly see or feel their tumour. This could have a powerful emotional impact for the few participants this affected, for example, Cathy could see and feel the tumour on her tongue growing rapidly larger which increased her fear: ‘The longer it was left, you could see it getting deeper and deeper, almost like eating the tongue away almost’ (Cathy, 40s, tongue, T2). Her concept of cancer as destructive appeared to be influenced heavily by seeing and feeling the cancer develop.

## Discussion

This study illustrates that people with head and neck cancers may generate mental images of their disease which may embody and influence their cognitive representation of their illness. To our knowledge, this is the first study to attempt to externalise and examine people’s internal mental images of their HNC. Mental images reflected beliefs about the identity, consequences (severity) and curability of the cancer, indicated in participants’ minds by its size, shape, and location. Greater size suggested a longer-established cancer, and a diffuse shape, such as having ‘roots’ that could spread, was perceived as more serious. Most participants perceived HNC as less severe than organ cancers, being distant from and therefore unlikely to affect major organs, e.g. the liver. Images also reflected beliefs about how cancer behaves: attacking or competing with healthy body structures. Specific beliefs related to Leventhal’s [22, 23] dimensions of disease consequences (e.g. its perceived severity), cure/control (e.g. the likelihood of treatment success) and physical identity (e.g. its appearance, how cancer behaves) were most salient in participants’ mental images.

Participants’ mental images could also affect their emotional response to (i.e. their emotional representation of) their illness producing anxiety or reassurance, depending on their associated beliefs about consequences and prognosis (cure/control). Indeed, the capacity of images to elicit emotions is widely acknowledged. For example, psychology studies investigating how the brain processes emotions routinely employ affective pictures [25, 26]; and personal images, such as foetal ultrasound scans, also produce an emotional response [27–29]. Linguistic cancer imagery and metaphors in health communication are known to affect people’s illness experiences [30, 31]. Cancer metaphors can potentially be harmful e.g., through the military language of war [32], but also protective e.g., through providing a less threatening means of perceiving and talking about cancer [33, 34]. There is less research into visual imagery. Visual images are heavily used in health promotion and cancer prevention materials, but a recent systematic review found a ‘disturbing lack of theory’ (p214) in research into visual imagery for skin cancer, and recommended further research [35]. Our findings indicate that language and imagery in early clinical encounters are highly influential in shaping mental images and related beliefs in people with HNC, which Harrow *et al*. [18] also found for breast cancer. Resulting mental images can cause significant distress, so clinicians should use imagery with caution.

### Study strengths and limitations

Our longitudinal study was rigorous and carefully conducted involving four senior qualitative researchers in addition to the primary researcher. We undertook a systematic analysis of interviews with a diverse sample of men and women with HNC. The longitudinal design, which allowed us to verify our understanding of participants’ first interviews during their second interview and to explore the stability or otherwise of their mental images of HNC, and independent coding of data by two to four authors, all contributed to rigour. The study samples were drawn from Scotland; future research could explore the applicability of our findings to other patient populations.

Based on a methodological pilot study with six people with HNC, two of their partners and four clinicians we anticipated at the outset of this study that people with HNC who had mental images of their cancer would be able to communicate them verbally or pictorially, yet many participants in the main study reported here refused to re-create their image. The provision of only coloured pencils, pens and white paper for image reproduction was also based on the pilot study findings, however, offering other creative media might have increased rates of image reproduction. The main study interviews suggested that concrete or abstract images could be frightening if they conveyed a diffuse picture of the cancer as opposed to it being ‘contained’; such fears, which had not been apparent in the pilot study, may have made participants reluctant to draw their cancer. For ethical reasons, participants’ refusal to draw their mental image was respected, particularly given the sensitive nature of the topic and the fact that participants were currently undergoing cancer treatment.

### Implications for clinical practice

Discussing internal mental images, verbally or pictorially, could allow clinicians access to the patient’s illness representation because images can reveal emotions, beliefs and expectations of outcome, and provide a common language with which to establish a therapeutic relationship [30]. Such discussions may also be valuable for exploring patients’ fears, particularly fears of cancer recurrence which are highly prevalent among HNC patients [36–38]. Clinicians may be unaware of patients’ expectations of disease and treatments [39, 40]; discussing patients’ images of their cancer may elicit any unrealistic expectations or inaccurate illness beliefs that could be managed through intervention. This may increase the patient’s overall satisfaction with care [41, 42] and foster a good clinician-patient relationship.

## Conclusions

Many people with HNC appear to have stable mental images of their cancer, generated early within clinical encounters. We theorise that attentive use of images in early consultations could avoid or minimise some distress, including fears of outcome or recurrence; and concrete or figurative images and language could be employed later to change perceptions and reduce distress.

## Supporting information

S1 File Topic guides for first and second interviews. (PDF)

## References

[1] Lang H, France E, Williams B, Humphris G, Wells M. The psychological experience of living with head and neck cancer: a systematic review and meta-synthesis. Psycho-Oncology. 2013 10.1002/pon.3343 23840037

[2] Chen A, Daly M, Vazquez E, Courquin J, Luu Q, Donald P, et al Depression Among Long-term Survivors of Head and Neck Cancer Treated With Radiation Therapy. JAMA Otolaryngol Head Neck Surg 2013;139(9):885–9. 10.1001/jamaoto.2013.4072 23949013

[3] Buchmann L, Conlee J, Hunt J, Agarwal J, White S. Psychosocial distress is prevalent in head and neck cancer patients. Laryngoscope. 2013;123(6):1424–9. Epub 2013/04/05. 10.1002/lary.23886 . 23553220

[4] Klein J, Livergant J, Ringash J. Health related quality of life in head and neck cancer treated with radiation therapy with or without chemotherapy: A systematic review Oral Oncology. 2014;50(4):254–62. 10.1016/j.oraloncology.2014.01.015 24559650

[5] Humphris G, Rogers S, McNally D, Lee-Jones C, Brown J, Vaughan D. Fear of recurrence and possible cases of anxiety and depression in oro-facial cancer patients. International Journal of Oral & Maxillofacial Surgery. 2003;32:486–91. 14759106

[6] Ghazali N, Cadwallader E, Lowe D, Humphris G, Ozakinci G, Rogers SN. Fear of recurrence among head and neck cancer survivors: longitudinal trends. Psychooncology. 2012 Epub 2012/03/28. 10.1002/pon.3069 . 22451036

[7] Luckett T, Britton B, Clover K, Rankin N. Evidence for interventions to improve psychological outcomes in people with head and neck cancer. A systematic review of the literature. Supportive Care in Cancer. 2011;19:871–81. 10.1007/s00520-011-1119-7 21369722

[8] Gould RV, Brown SL, Bramwell R. Psychological adjustment to gynaecological cancer: Patients' illness representations, coping strategies and mood disturbance. Psychology & Health 2010;25(5):633–46. 20204950 10.1080/08870440902811163

[9] Paddison JS, Booth RJ, Cameron LD, Robinson E, Frizelle FA, Hill AG. Fatigue After Colorectal Surgery and Its Relationship to Patient Expectations Journal of Surgical Research. 2009;151:145–52. 10.1016/j.jss.2008.01.030 18468632

[10] McCorry NK, Dempster M, Quinn J, Hogg A, Newell J, Moore M, et al Illness perception clusters at diagnosis predict psychological distress among women with breast cancer a 6 months post diagnosis. Psychooncology. 2013;22(3):692–8. 10.1002/pon.3054 22389291

[11] Corter A, Findlay M, Broom R, Porter D, Petrie K. Beliefs about medicine and illness are associated with fear of cancer recurrence in women taking adjuvant endocrine therapy for breast cancer British Journal of Health Psychology 2013;18:168–81. 10.1111/bjhp.12003 23134580

[12] Scharloo M, de Jong R, Langeveld T, van Velzen-Verkaik E, den Akker M-o, Kaptein A. Illness cognitions in head and neck squamous cell carcinoma: predicting quality of life outcome. Supportive Care in Cancer. 2010;18:1137–45. 10.1007/s00520-009-0728-x 19718524 PMC2910308

[13] Ashley L, Marti J, Jones H, Velikova G, Wright P. Illness perceptions within 6 months of cancer diagnosis are an independent prospective predictor of health-related quality of life 15 months post-diagnosis. PsychoOncology. 2015;24: 1463–70. 10.1002/pon.3812 25946704

[14] Broadbent E, Ellis CJ, Gamble G, Petrie KJ. Changes in Patient Drawings of the Heart Identify Slow Recovery After Myocardial Infarction Psychosomatic Medicine. 2006;68(6):910–3. 10.1097/01.psy.0000242121.02571.10 17079705

[15] Reynolds L, Broadbent E, Ellis CJ, Gamble G, Petrie KJ. Patients’ drawings illustrate psychological and functional status in heart failure. Journal of Psychosomatic Research. 2007;63:525–32. 10.1016/j.jpsychores.2007.03.007 17980226

[16] Broadbent E, Petrie KJ, Ellis CJ, Ying J, Gamble G. A picture of health—myocardial infarction patients' drawings of their hearts and subsequent disability A longitudinal study. Journal of Psychosomatic Research. 2004;57: 583–7. 10.1016/j.jpsychores.2004.03.014 15596165

[17] Cameron LD. Illness risk representations and motivations to engage in protective behavior: The case of skin cancer risk. Psychology & Health. 2008;23(1):91–112. 25159909 10.1080/14768320701342383

[18] Harrow A, Wells M, Humphris G, Taylor C, Williams B. Seeing is believing, and believing is seeing’: An exploration of the meaning and impact of women’s mental images of their breast cancer and their potential origins. Patient Education and Counselling 2008;73:339–46. 10.1016/j.pec.2008.07.014 18722745

[19] Morgan S, Roberts P, Stahlschmidt J. Editorial: What colour is my cancer? Engaging teenage and young adult patients with their disease. European Journal of Cancer. 2008;44:1483–4. 10.1016/j.ejca.2008.04.001 18550359

[20] Llewellyn C, McGurk M, Weinman J. Illness and treatment beliefs in head and neck cancer: is Leventhal's common sense model a useful framework for determining changes in outcomes over time? Journal of Psychosomatic Research. 2007;63(1):17–26. 10.1016/j.jpsychores.2007.01.013 17586334

[21] Smith JA, Jarman M, Osborn M. Doing interpretative phenomenological analysis In: Murray M, Chamberlain K, editors. Qualitative Health Psychology: Theories and Methods. London: SAGE; 1999 p. 218–40.

[22] Leventhal H, Phillips LA, Burns E. The Common-Sense Model of Self-Regulation (CSM): A dynamic framework for understanding illness self-management. Journal of behavioral medicine. 2016;39(6):935–46. 10.1007/s10865-016-9782-2 27515801

[23] Leventhal H, Brissette I, Leventhal E. The common-sense model of self-regulation of health and illness In: Cameron L, Leventhal H, editors. The Self-Regulation of Health and Illness Behaviour. London: Routledge; 2003 p. 42–65.

[24] Scottish Government. Scottish Index of Multiple Deprivation 2012. A National Statistics Publication for Scotland, 18 12 2012 2012. Available from: http://simd.scotland.gov.uk/publication-2012/.

[25] Bradley M, Codispoti M, Cuthbert BN, Lang PJ. Emotion and Motivation I: Defensive and Appetitive Reactions in Picture Processing Emotion 2001;1(3):276–98. 12934687

[26] Olofsson JK, Nordin S, Sequeira H, Polich J. Affective picture processing: An integrative review of ERP findings. Biological Psychology. 2008;77:247–65. 10.1016/j.biopsycho.2007.11.006 18164800 PMC2443061

[27] Harpel T, Hertzog J. “I Thought My Heart Would Burst”: The Role of Ultrasound Technology on Expectant Grandmotherhood. Journal of Family Issues. 2010;31(2):257–74.

[28] Ji E, Pretorius D, Newton RU, Uyan K, Hull A, Hollenbach K, et al Effects of ultrasound on maternal-fetal bonding: a comparison of two- and three-dimensional imaging. Ultrasound in Obstetrics and Gynaecology. 2005;25(5):473–7. 10.1002/uog.1896 15846757

[29] Campbell S, Reading AE, Cox DN, Sledmere CM, Mooney R, Chudleigh P, et al Ultrasound scanning in pregnancy: The short-term psychological effects of early real-time scans. Journal of Psychosomatic Obstetrics & Gynecology. 1982;1(2):57–61. 10.3109/01674828209081226

[30] Harrington K. The use of metaphor in discourse about cancer: a review of the literature. Clinical Journal of Oncology Nursing. 2012;16(4):408–12. 10.1188/12.CJON.408-412 22842692

[31] Appelton L, Flynn M. Searching for the new normal: Exploring the role of language and metaphors in becoming a cancer survivor. European Journal of Oncology Nursing. 2014;18(4):378–84. 10.1016/j.ejon.2014.03.012 24785792

[32] Sontag S. Illness as Metaphor. New York: Anchor; 1978.

[33] Penson RT, Schapira L, Daniels KJ, Chabner BA, Lynch TJ. Cancer as metaphor. The Oncologist 2004;9:708–16. 10.1634/theoncologist.9-6-708 15561814

[34] Lanceley A, Clark JM. Cancer in other words? the role of metaphor in emotion disclosure in cancer patients. British Journal of Psychotherapy 2013;29 (2):182e201. 24748706 10.1111/bjp.12023 PMC3985809

[35] McWhirter J, Hoffman-Goetz L. Visual images for skin cancer prevention: a systematic review of qualitative studies. Journal of Cancer Education. 2012;27(2):202–16. 10.1007/s13187-012-0355-y 22481615

[36] Humphris GM, Ozakinci G. Psychological responses and support needs of patients following head and neck cancer. Int J Surg. 2006;4(1):37–44. Epub 2007/04/28. 10.1016/j.ijsu.2005.12.004 . 17462312

[37] Rogers S, Scott B, Lowe D, Ozakinci G, Humphris G. Fear of recurrence following head and neck cancer in the outpatient clinic. European Archives of Otorhinolaryngology. 2010;267:1943–9. 10.1007/s00405-010-1307-y 20582704

[38] Wells M, Cunningham M, Lang H, Swartzman S, Philp J, Taylor L, et al Distress, concerns and unmet needs in survivors of head and neck cancer: a cross-sectional survey European Journal of Cancer Care 2015;24(5). 10.111/ecc.12370 26250705

[39] List M, Stracks J, Colangelo L, Butler P, Ganzenko N, Lundy D, et al How do head and neck cancer patients prioritize treatment outcomes before initiating treatment? Journal of Clinical Oncology. 2000;18(4):877–84. 10.1200/JCO.2000.18.4.877 10673531

[40] Tschiesner U, Sabariego C, Linseisen E, Becker S, Stier-Jarmer M, Cieza A, et al Priorities of head and neck cancer patients: a patient survey based on the brief ICF core set for HNC. Eur Arch Otorhinolaryngol. 2013;270(12):3133–42. Epub 2013/04/02. 10.1007/s00405-013-2446-8 . 23543319

[41] Llewellyn C, McGurk M, Weinman J. How satisfied are head and neck cancer (HNC) patients with the information they receive pre-treatment? Results from the satisfaction with cancer information profile (SCIP). Oral Oncology. 2006;42:726–34. 10.1016/j.oraloncology.2005.11.013 16529976

[42] Winterling J, Sidenvall B, Glimelius B, Nordin K. Expectations for the recovery period after cancer treatment–a qualitative study. European Journal of Cancer Care. 2009;18(6):585–93. 10.1111/j.1365-2354.2008.00933.x 19686270

