# Supplementary material for: The existence and importance of patients’ mental images of their head and neck cancer: A qualitative study
Source: PLoS One. 2018 Dec 31;13(12):e0209215. doi: 10.1371/journal.pone.0209215 (PMC6312291; doi:10.1371/journal.pone.0209215)
Supplement: S1 File — (PDF) [file pone.0209215.s001.pdf]

S1 file

TOPIC GUIDE FOR INTERVIEW 1

- Thank you for participating
- Any questions?
- Confidentiality
- Refuse to answer
- Consent form
- GP letter – take address
- Demographics details
- Disease details

…………………………………………………………………………………………………….

- Your experience of cancer so far.
  - Prior experience – own or familial
  - Current cancer – symptoms, diagnosis, cause
- What does cancer mean to you
  - What is it – Could you describe cancer to me as you understand it (identity)
  - What does it do – its physical action, the consequences
  - What are its characteristics – what is it made of, how does it originate
  - How do you feel about it
  - Where does this understanding come from – previous knowledge, HPs
- What do you think is going on inside your body right now?
  - Are the disease effects local or far-reaching
  - Location
  - Behaviour
  - If untreated?
- Mental Image
  - Ever thought about what it looks like?
  - Do you have a picture of it in your mind's eye?
- Tell me a bit about the treatment you're going to be having
  - Understanding - Could you describe this treatment to me as you understand it
  - Fears/expectations
  - What will it do – its action on the cancer
  - What happens to the cancer?
- Exposure to imagery
  - Visual language
  - Scans, nasoendoscope, HP language
- Impact of cancer on your life
  - Emotionally – Work – Relationships – Leisure

…………………………………………………………………………………………………….

- Anything you would like to add?
- Support Services Booklet
- Arranging 2nd interview
- Any questions?
- Thanks

TOPIC GUIDE FOR INTERVIEW 2

- Thank you for participating, settle in, and warm up conversation.
- Recap last interview and answer questions.
- Tell me a bit about how things have been going (the experience)
- And how did your treatment go
    - Visual language
    - Practical experience
    - Its action/aim
    - Side effects – expected and actual
- What do you think is going on inside your body now?
    - With the cancer
    - After the treatment – i.e. in terms of affected tissues etc
- Mental image of the cancer
    - This is the drawing you produced last time – is it still relevant
    - Any changes in the way you visualise it
    - Any other images
    - What kind of feelings did/does this image bring up
    - How do you now envision the site where the cancer has been
- Tell me about how your beliefs/understanding/expectations have changed over time
    - What cancer means
    - What the treatment is like
- Thoughts about the future
    - How will your body be – i.e. as before, forever changed etc
    - What does recovery mean
    - Fears/expectations
- Thanks, questions, warm down conversation.
